# The involvement of endoplasmic reticulum stress response in immune dysfunction of dendritic cells after severe thermal injury in mice

**DOI:** 10.18632/oncotarget.14764

**Published:** 2017-01-03

**Authors:** Xiao-Mei Zhu, Ning Dong, Yan-Bo Wang, Qing-Hong Zhang, Yan Yu, Yong-Ming Yao, Hua-Ping Liang

**Affiliations:** ^1^ State Key Laboratory of Trauma, Burns and Combined Injury, Research Institute of Surgery, Daping Hospital, The Third Military Medical University, Chongqing, China; ^2^ Department of Microbiology and Immunology, Trauma Research Center, First Hospital Affiliated to the Chinese PLA General Hospital, Beijing, China

**Keywords:** endoplasmic reticulum stress, dendritic cell, immune dysfunction, burns, sepsis, Pathology Section

## Abstract

Suppressed adaptive immune function is one of the major concerns responsible for the development of opportunistic infections and subsequent sepsis with high mortality in severe burns. Endoplasmic reticulum stress (ERS) is the endogenous self-protective mechanism, and it plays an important role in almost every process of living by regulating the balance between homeostasis and apoptosis. The current study investigated the involvement of ERS in the pathogenesis of dysfunction of dendritic cells (DCs) in burn mice. Our results show a significant ERS response in splenic DC after burn injury. Treatment with salubrinal (Sal, reported to protect cells against ERS-induced apoptosis.) decrease the apoptotic rate of DC induced by burns, and promote maturation and activation of DC, as well as the ability to promote T cell proliferation and polarization towards Th1 immunity (all *P*<0.05). Gene silence of XBP-1 (key molecular in ERS response) results in the increased apoptosis and suppressed phenotypical maturation of splenic DC in burn mice. These results show that the excessive ERS is essential for immunosuppression during severe thermal injury. XBP-1 plays a pivotal role in DC functional immunomodulation in burn mice. Inhibition of apoptotic ERS response benefits mice from major burns.

## INTRODUCTION

Burns cause varying degrees of pain, blisters, swelling, and skin loss. Deep, extensive burns can cause serious complications, with thorny issues like multi-resistance infections and high mortality [1, 2]. Host response associated with suppressed adaptive immune function might be one of the main concerns responsible for the development of opportunistic infections and subsequent sepsis with high mortality in major burns [3–6]. Dendritic cells (DCs), the most important potent antigen-presenting cells (APCs), are unique in their capacity to prime naive T cells and initiate immune response [7–9]. Several reports and our previous study have demonstrated that burn injury induced impairments in immunobiology of DCs, resulting in suppression of adaptive immune response [10, 11]. Patenaude also reported that burn injury caused a state of reprogramming DC subsets that were no longer capable of responding adequately to any subsequent challenge [12]. Moreover, a profound loss in the number of DCs, which mostly result from the apoptosis, was observed in spleen in burn injury as well as in sepsis [12–14]. Functions of DCs could be influenced by endogenous signals and exogenous stimuli. Given the critical role of DCs in immune system, we believed that the DC’ tug-of-war to maintain functional integrity in the post-injury period was of great significance in the setting of severe burns. In the present study, we are focused on endoplasmic reticulum (ER) as a stress-responsive organelle in splenic DCs.

ER is a very versatile organelle in eukaryotic cells. By providing a stable microenvironment for the synthesis of metabolites and secreted proteins, ER functions as an intracellular stress sensory organelle and, accordingly, initiates as well as regulates adaptive responses to environmental abiotic and biotic stress [15]. Different stress stimuli, e.g., hypoxia, viral replication, abnormal proteins and starvation, all ultimately lead to accumulation of unfolded or misfolded proteins in the lumen of the ER [16–18] and activation of unfolded protein response (UPR), resulting in a condition referred as ER stress (ERS) [19]. Three proteins including PERK, IRE-1α, and ATF6 located in the ER membrane are known as stress sensor to respond to ERS stimuli by triggering the intracellular signal transduction pathways, and activating several transcription factors serve for cell survival during the early stages of stress and for apoptotic cell death during acute or chronic stress.

Proper function of ER is vital for the establishment of an effective immune system. A series of studies had proved that ERS was involved in activation and function modulation of immune cells [20–23]. However, it remains less studied as to how ERS may influence or alter cellular activities and functions in innate and adaptive immunity in severe burns. The current study was conducted to investigate the involvement of ERS in DC dysfunction after severe thermal injury, in order to provide intensive insights into endogenous sources of cellular stress as the focus of elucidation of regulatory mechanisms of immunosuppression in severe burns.

## RESULTS

### Dysfunction of splenic DCs in mice with thermal injury

**Figure 1:**
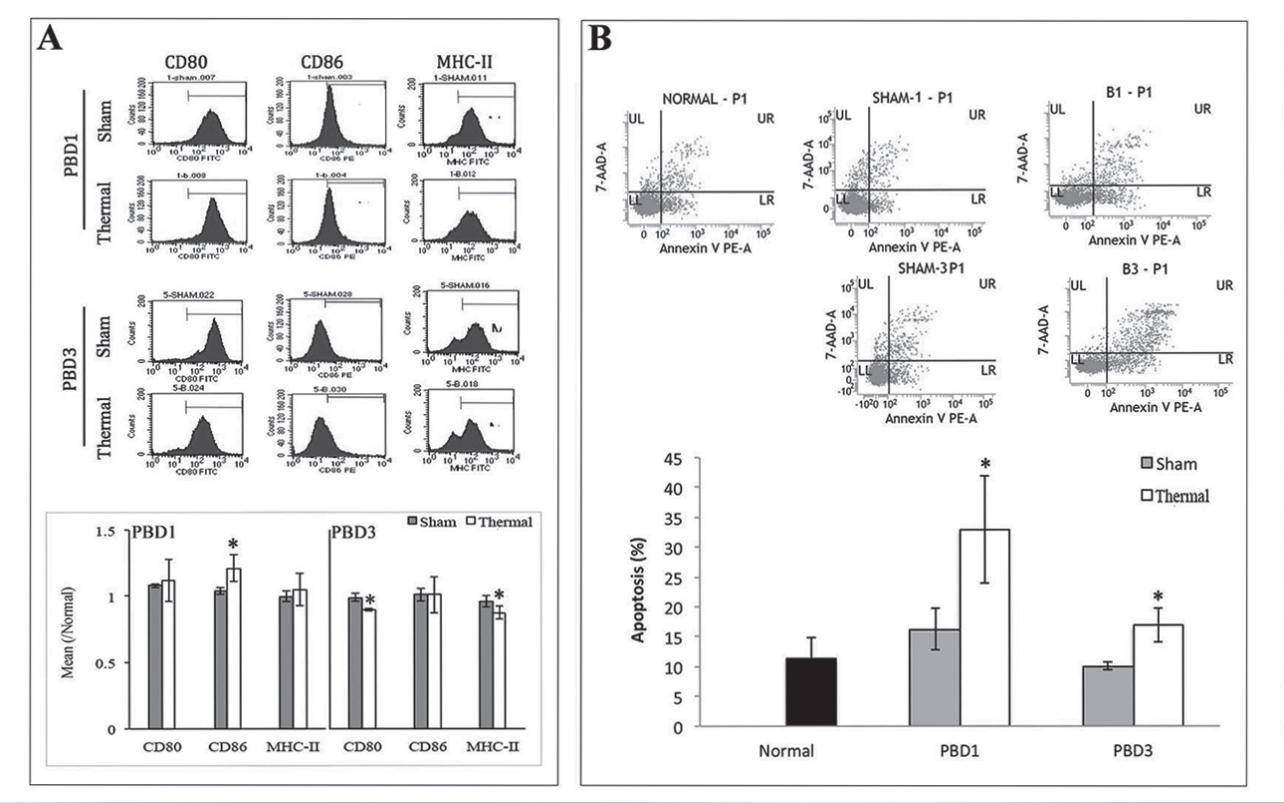
Thermal injury induced dysfunction of splenic DC in mice Splenic DCs were isolated with CD11c MicroBeads from burn mice. A. The expression of co-stimulatory molecules including CD80, CD86 and MHC-II on surface of DCs was determined by flow cytometry and analyzed by the ratios to normal value in the same batch. B. Apoptosis rate of splenic DCs was determined by flow cytometric analysis. The apoptosis rates were assessed using the sum of area UR (Annexin-V/7-AAD indicates late apoptotic cells) and area LR (Annexin-V^+^/7-AAD^-^ indicates early apoptotic cells). Results were shown in mean ± SD (*n* = 6). * Statistically significant difference when compared with sham group at the same time point (*p* < 0.05).

Model of 15% TBSA fullthickness scalded mice was used as experimental animal and CD11c^+^ DCs were collected with MicroBeads (Miltenyi Biotech, Bergisch Gladbach, Germany). Phenotypic and functional changes in splenic DCs of burn mice were analyzed on post-burn days (PBD) 1-3. As shown in Figure 1A, DC from spleens of burn mice expressed low levels of CD80, strongly enhanced levels of CD86 during PBD 1-3, and slightly enhanced levels of MHC-II on PBD 1, demonstrating abnormal maturation when compared to DC from sham-injured mice. And significant apoptosis was found in splenic DCs in burn-injured mice (Figure 1B).

### ERS response in splenic DCs in thermal injury

**Figure 2:**
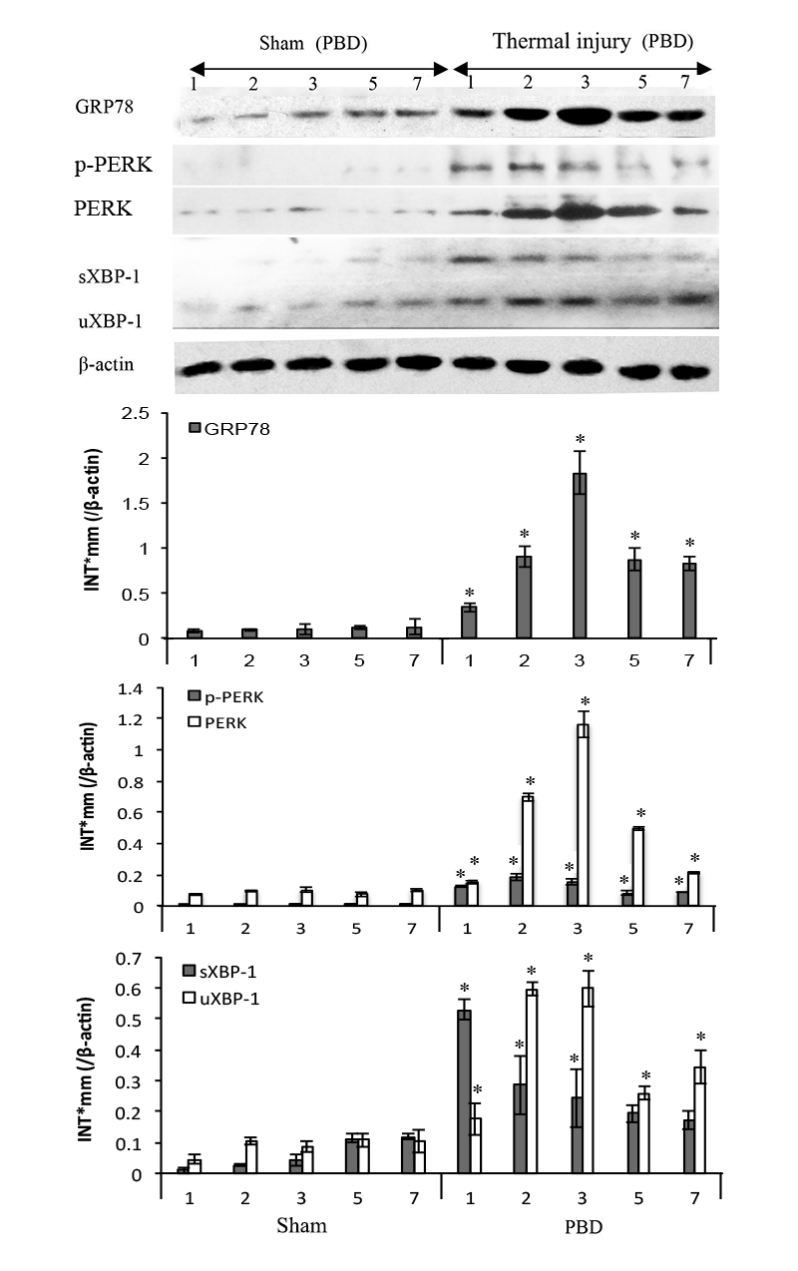
Thermal injury induced ERS response in splenic DC in mice Expressions of GRP78, PERK, p-PERK, XBP-1 (including full length and spliced form) in DC were assessed by Western blot analysis. Representative images from the same membrane were shown. Data were shown in mean ± SD (*n* = 4). * Statistically significant difference when compared with sham group at the same time point (*p* < 0.05). P.s uXBP-1: full length XBP-1 (unactivated). sXBP-1: spliced XBP-1(activated)

To assess the expression levels of ER chaperonins in DCs in thermal injury, total proteins were extracted with experimental procedure as described, and Western blot analysis was performed. As shown in Figure 2, the expression or activation levels of ERS markers in splenic DCs were markedly up-regulated with thermal injury. Protein levels of GRP78, total and phosphorylated PERK, as well as activation of transcription factor XBP-1 increased in burn mice on PBD1-3 and remained high level till PBD7 (all *P* < 0.05). Phosphorylation of PERK and activation of transcription factor XBP-1 were especially noticeable on PBD 1 (all *P* < 0.05).

### Counteract ERS-induced apoptosis with Salubrinal protects mice from severe thermal injury

**Figure 3:**
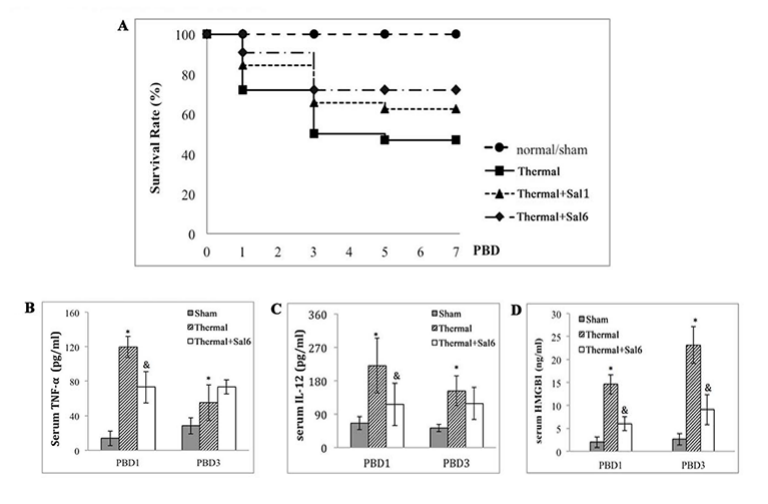
Protective effects of Sal on thermal injured mice **A.** Survival rate of burn mice were monitored for over 7 d (= 48). Sal (1mg/kg) was administered at 1 h (Sal1) or 6 h (Sal6) after thermal injury. **B.-D.** Inflammatory mediators in serum of thermal mice model were assessed with ELISA (*n* = 6). Sal (1mg/kg) was administered at 6 h after thermal injury. * Statistically significant difference when compared with sham group at the same time point (*p* < 0.05). & Statistically significant difference when compared with thermal-injured group at the same time point (*p* < 0.05).

We treated mice with Salubrinal (Sal) to investigate the involvement of ERS response in thermal injury. Sal was reported to protect cells against ERS-induced apoptosis but not apoptotic stimuli unrelated to ERS [24]. Survival rate of thermal injured mice treated with Sal was observed for over 7 days. As shown in Figure 3, Sal treatment post-burn provided significant protective effect, showing a favorable survival rate when compared with untreated burn group (*n* = 48, *P* < 0.05). The survival rate of Sal6 group (Sal administrated at 6 h after injury) was higher than that of Sal1 group (Sal administrated at 1 h after injury) although the statistical difference was not significant (58.33% v.s. 70.83%) (*P* = 0.175). We decided to use Sal at 6 h after burn in the subsequent experiments.

The inflammatory response of burn mice treated with Sal at 6 h post-burn was also evaluated. We assessed the levels of inflammatory cytokines including IL-12, TNF-α, and HMGB1 (an important late inflammatory cytokine) in serum of mice model. As shown in Figure 3, treatment with Sal resulted in a significant lowering in IL-12, TNF-α levels on PBD1 (Figure 3B, 3C), and HMGB1 levels on PBD3 (Figure 3D) (all *P* < 0.05).

### Effect of Sal on immune function of DCs after thermal injury

**Figure 4:**
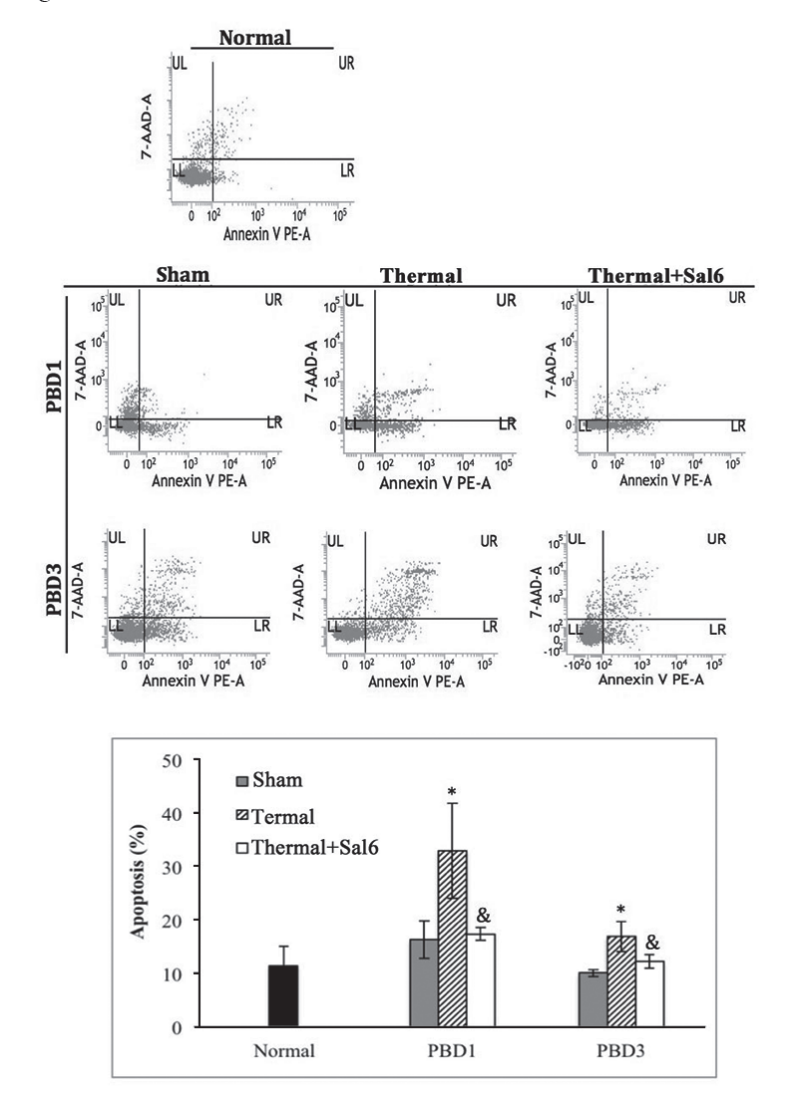
Sal-treatment decreased the apoptosis rate of splenic DCs in thermal injured mice Splenic DCs were isolated with CD11c^+^ MicroBeads from mice model. Apoptosis rate of splenic DCs was determined by flow cytometric analysis. Sal (1mg/kg) was administered at 6 h after thermal injury. The apoptosis rates were assessed using the sum of area UR (Annexin-V^+^/7-AAD^-^ indicates late apoptotic cells) and area LR (Annexin-V^+^/7-AAD^-^ indicates early apoptotic cells). Results were shown in mean ± SD (n = 6). * Statistically significant difference when compared with sham group at the same time point (*p* < 0.05). & Statistically significant difference when compared with thermal-injured group (*p* < 0.05).

**Figure 5:**
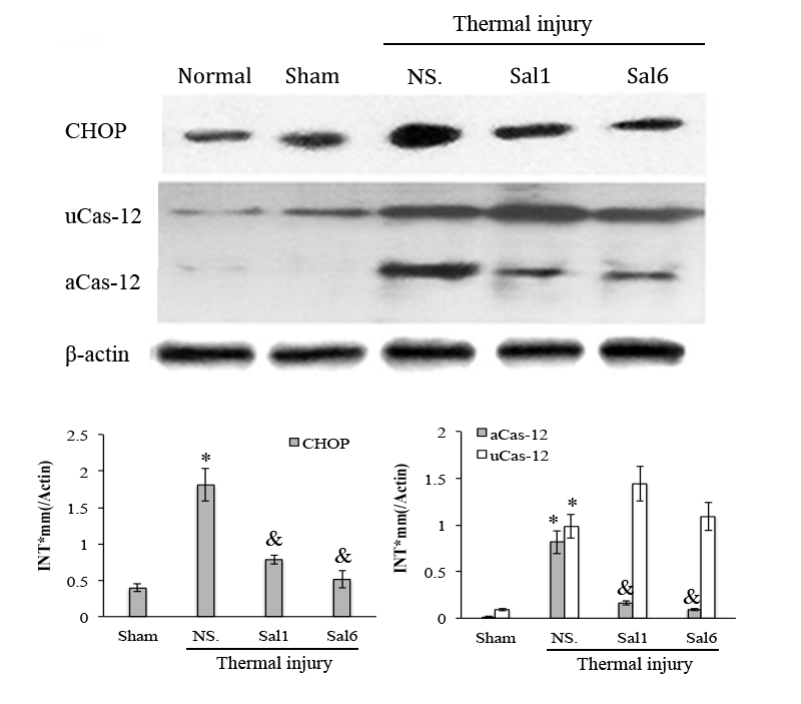
Sal-treatment relieved the ERS response in splenic DCs in thermal injured mice Splenic DCs were isolated from mice model on PBD 1. The expression of ERS markers in splenic DCs was detected with Western blot analysis. Sal (1mg/kg) was administered at 6 h after thermal injury. Representative images from the same membrane were shown. Data were shown in mean ± SD (*n* = 4). * Statistically significant difference when compared with Sham group (*p* < 0.05). & Statistically significant difference when compared with thermal-injured group (*p* < 0.05). P.s uCas-12: unactivated caspase-12. aCas-12: activated caspase-12.

To study the exact effect of Sal-treatment on DC’s immune function, we measured the apoptosis and functional phenotype of splenic DCs in burn mice. As shown in Figure 4, scald mice treated with Sal had significantly lower rate of apoptosis in splenic DCs than untreated burn mice (*P* < 0.05). The expression level of CHOP and the activation caspase-12, two important factors in ERS-related apoptosis signaling pathway, were both decreased in DCs from thermal injured mice after Sal administration (all *P* < 0.05) (Figure 5).

**Figure 6:**
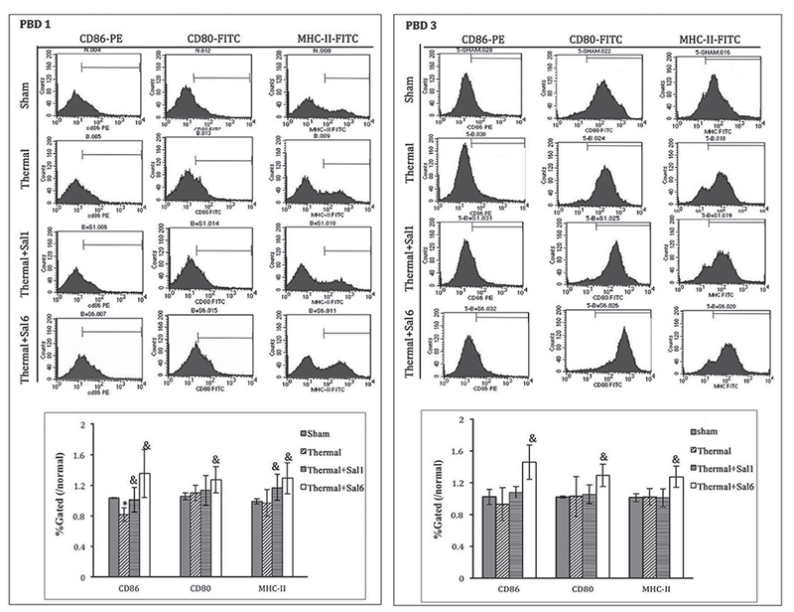
Sal-treatment promote the phenotypic maturation of splenic DC in thermal injured mice Splenic DCs were isolated with CD11c^+^ MicroBeads from mice model. The expression of co-stimulatory molecules including CD80, CD86 and MHC-II on surface of DCs was determined by flow cytometry and analyzed by the ratios to normal value in the same batch. Sal (1mg/kg) was administered at 1 h (Sal1) or 6 h (Sal6) after thermal injury. Results were shown in mean ± SD (*n* = 6). * Statistically significant difference was found when compared with sham group at the same time point (*p* < 0.05). & Statistically significant difference when compared with thermal-injured group (*p* < 0.05).

**Figure 7:**
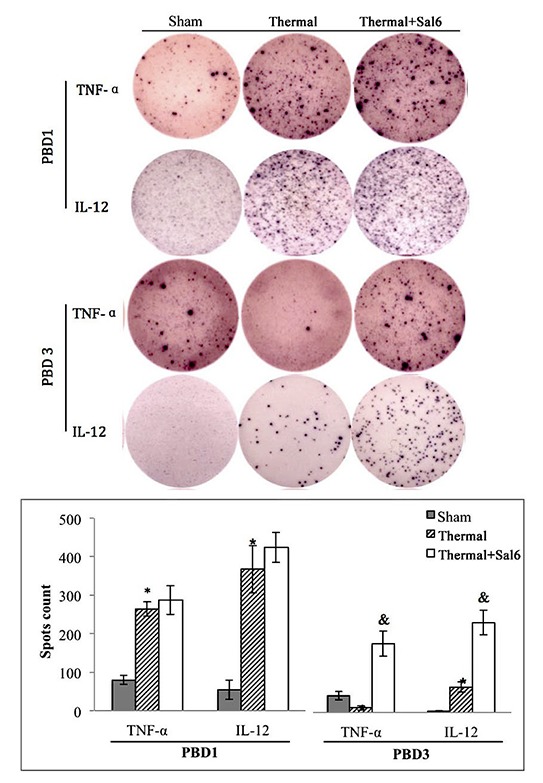
Sal treatment improved the secretion ability of splenic DCs in thermal injured mice Splenic DCs were isolated with CD11c^+^ MicroBeads from mice model and plated to the microplate of ELISpot Assay (2*10^5^cell/well) and cultured overnight (18 hours). Spots were analyzed with ELISpot reader system. Sal (1mg/kg) was administered at 6 h after thermal injury. Results were shown in mean ± SD (*n* = 6). * Statistically significant difference when compared with sham group at the same time point (p < 0.05). & Statistically significant difference when compared with thermal-injured group (*p* < 0.05).

Moreover, treatment with Sal markedly enhanced the maturation and activation of DC after thermal injury. Levels of CD80, CD86, and MHC-II on the surface of DC were elevated in burn mice treated with Sal (Figure 6). In addition, ELISpot assay was employed to measure the secretion ability of individual DC. As shown in Figure 7, release of IL-12 and TNF-α, the markers of DC maturation and key mediators for T cell modulation, was increase on PBD1 but depressed on PBD 3 in burn mice. Treatment with Sal after injury could improve the secretion ability of DCs slightly on PBD1and significantly on PBD 3 (Figure 7).

**Figure 8:**
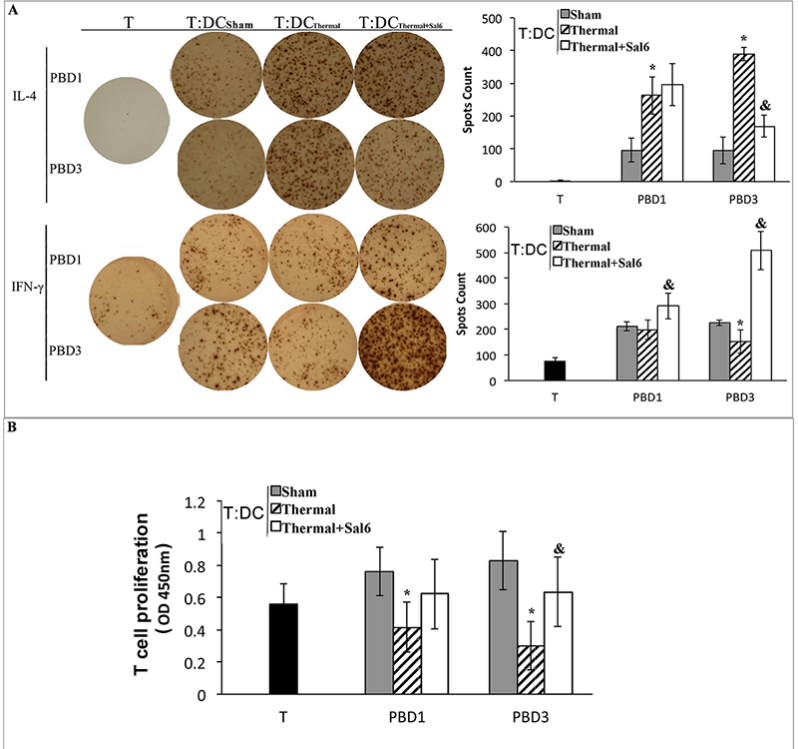
Sal-treatment ameliorated regulating ability of splenic DCs on T cell polarization and proliferation T cells were isolated from normal mice, stimulated with soluble CD3 (1 μg/ml) and soluble CD28 (5 μg/ml) for 24 hours. Splenic DCs were isolated with CD11c^+^ MicroBeads from mice model and co-cultured with T mentioned above at a DC: T ratio of 1: 200 (the concentration of T was 2*10^5^cell/well) and cultured for 3 days. Production of IFN-γ and IL-4 was assessed with ELISpot Assay A. T cell proliferation was detected with CCK-8 cell counting kit B. Sal (1mg/kg) was administered at 6 h after thermal injury. Results were shown in mean ± SD (*n* = 6). * Statistically significant difference when compared with sham group at the same time point (*p* < 0.05). & Statistically significant difference when compared with thermal-injured group (*p* < 0.05).

We further investigated the amelioration of DCs as the regulator of CD4^+^ T cell polarization after treatment with Sal. DCs isolated from burn mice with or without Sal treatment were co-cultured with CD4^+^ T cells isolated from normal controls. The proportion of cytokine-producing cells was assessed with ELISpot assay. T cell proliferation was assessed with CCK-8 kit. As shown in Figure 8A, co-culture with splenic DCs from Sal-treated burn mice on PBD 3 could induce Th1 polarization, although this trend was not significant on PBD1. The ability of T cells to secret IFN-γ was obviously increased. And the level of IL-4 was significantly reduced (all *P* < 0.05) (Figure 8A). The proliferation rate of T cells was also higher when they were co-cultured with DCs from Sal-treated scald mice on PBD3 than that with DCs from untreated burn mice (Figure 8B) (*P* < 0.05).

### Gene silence of XBP-1 impaired splenic DC’s immune function

**Figure 9:**
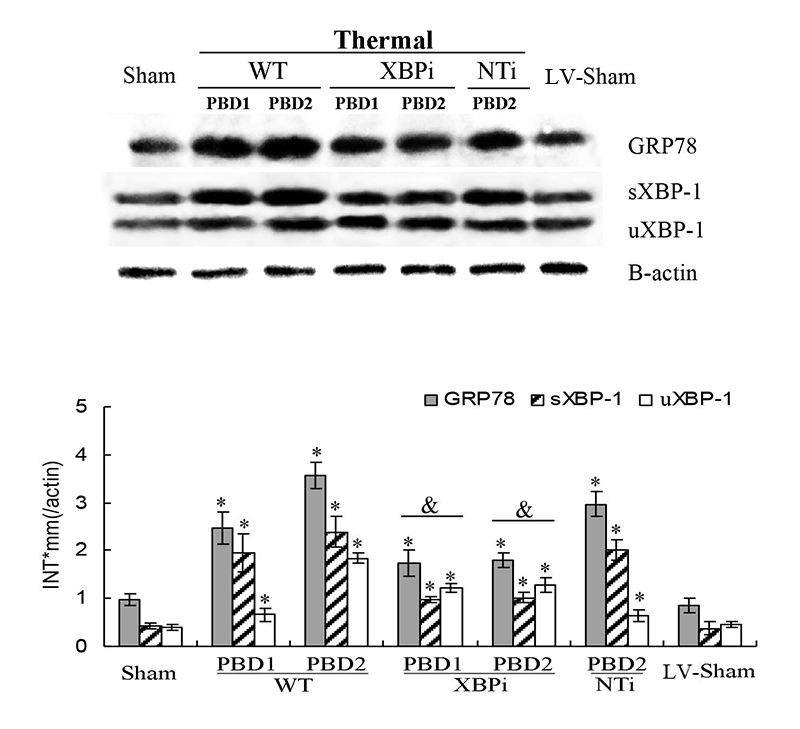
Effects of XBP-1 gene silence in splenic DC in thermal injured mice model Mice transfected with XBPi-LV (3.5×10TU per mice) were subjected to thermal injury. Expressions of GRP78 and XBP-1 (including full length and spliced form) in splenic DCs were assessed by Western blot analysis. Representative images from the same membrane were shown. Data were shown in mean ± SD (n = 4). * Statistically significant difference when compared with sham group at the same time point (*p* < 0.05). Statistically significant difference when compared with thermal injured mice in WT or NTi group (*p* < 0.05).

XBP-1 was proved to be a key factor in the ERS response [21]. A lentiviral vector (LV) was constructed for XBP-1 gene RNA interference (XBPi), and a nontargeting stem-loop oligonucleotide (NTi) was used as sham-silence control. Mice were injected with RNAi-LV *via* tail vein and raised for 2 weeks before subsequent experiments. As shown in Figure 9, protein levels of XBP-1 (both full and spliced form) in splenic DC of XBPi-burn mice were significantly lower than that of wild type (WT)-burn mice (all *P* < 0.05), indicating a confirmed interference efficiency of XBPi *in vivo*. The difference between Sham and LV-Sham as well as that between WT-burn mice and NTi-burn mice was not obvious, indicating little effect of LV itself to mouse.

**Figure 10:**
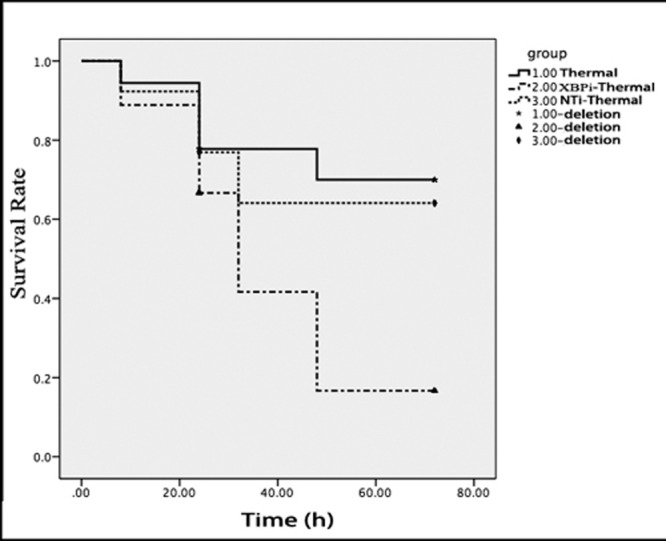
Gene silence of XBP-1 lowered the survival rate of thermal-injured mice on PBD1 Totally18 normal mice, 18 mice injected with XBPi-LV and 13 mice injected with NTi-LV were subjected to thermal injury in this experiment. Four live mice on PBD1 and two on PBD3 were picked randomly from every group and killed for sample collection. Log-rank test was used to compare the survival times of the rest mice till 72 hours after thermal injury (*p* = 0.041). The survival rate of XBPi- group was markedly lower than that of normal mice (*p* = 0.017).

Results of subsequent experiment showed that the gene silence of XBP-1 weakened the stress-resistant ability of mice to burn injury. The survival rate of XBPi-transfected mice subjected to scald injury on PBD 1-3 was lower than that of WT or NTi-transfected mice (*n* = 18, *P* < 0.05) (Figure 10).

**Figure 11:**
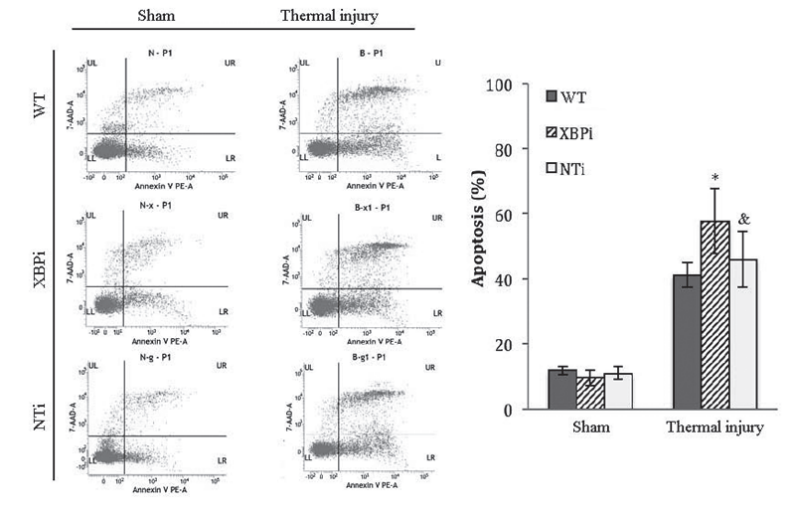
XBPi-transfection increased the apoptosis rate of splenic DCs in burn mice on PBD1 Splenic DCs were isolated with CD11c^+^ MicroBeads from mice model on PBD1. Apoptosis rate of splenic DCs was determined by flow cytometric analysis. The apoptosis rates were assessed using the sum of area UR (Annexin-V^+^/7-AAD^-^ indicates late apoptotic cells) and area LR (Annexin-V^+^/7-AAD^-^ indicates early apoptotic cells). Results were shown in mean ± SD (*n* = 4). * Statistically significant difference when compared with sham group at the same time point (< 0.05). & Statistically significant difference when compared with thermal-injured group (*p* < 0.05).

**Figure 12:**
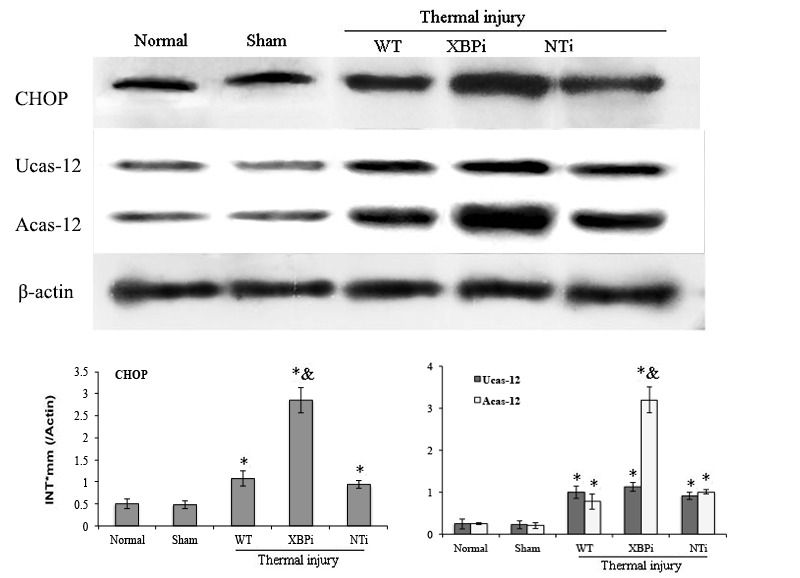
Gene silence of XBP-1 up-regulated the activation of ERS-related apoptosis pathway in splenic DCs in thermal injury Splenic DCs were isolated from mice model on PBD 1. The expression of CHOP and activation of caspase-12 in splenic DCs was detected with Western blot analysis. Representative images from the same membrane were shown. Data were shown in mean ± SD (*n* = 4). * Statistically significant difference when compared with Sham group (*n* < 0.05). & Statistically significant difference when compared with WT or NTi group (*p* < 0.05).

The influence of XBP-1 silencing on the functional status of DC was investigated in mice on PBD 1-3. XBPi transfection significantly elevated the apoptotic rate of splenic DCs in burn mice on PBD1, but had no marked effect on DC apoptosis in mice without burn injury (Figure 11). Levels of CHOP expression and cas-12 activation (key factors in the ERS-related apoptotic pathway) induced by burn injury were obviously higher in DCs from XBPi-transfected mice than WT mice (*P* < 0.05) (Figure 12).

**Figure 13:**
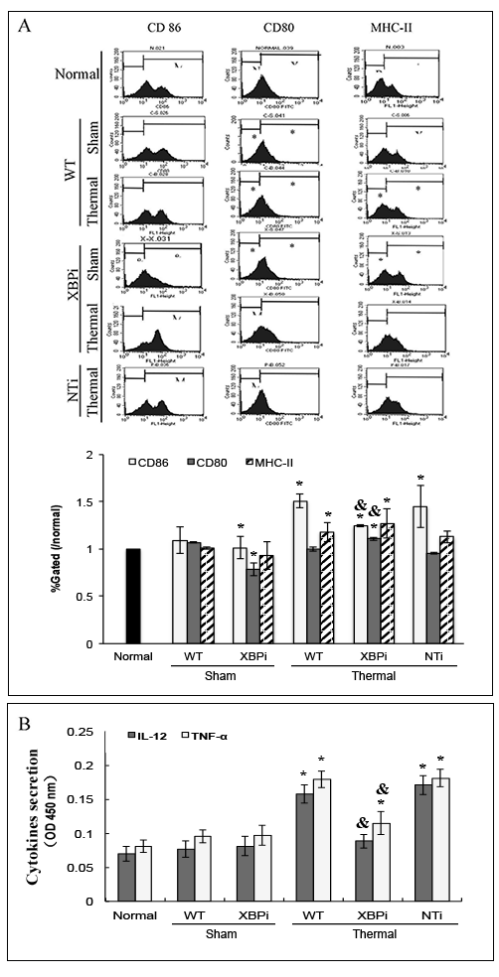
XBPi-transfection affected the surface phenotype and cytokines secretion of splenic DC in mice Splenic DCs were isolated with CD11c^+^ MicroBeads from mice model on PBD1 and cultured at 37°C in a humidified atmosphere with 5% CO2 overnight. **A.** The expression of co-stimulatory molecules including CD80, CD86 and MHC-II on surface of DCs was determined by flow cytometry and analyzed by the ratios to normal value in the same batch. **B.** Protein level of TNF-α and IL-12 in cell culture medium was determined with ELISA. Results were shown in mean ± SD (*n* = 4). * Statistically significant difference when compared with sham group (*p* < 0.05). & Statistically significant difference when compared XBPi with WT or NTi group (*p* < 0.05).

In addition, the surface phenotype of splenic DC was significantly affected by XBPi-transfection. Expressions of costimulatory molecules including CD80 and CD86, and MHC-II on surface of DCs were reduced in normal mice with XBP-1 silence (Figure 13A). When the XBPi-mice were subjected to burn injury, levels of these phenotypic markers increased slightly, but they still lower than that in WT or NTi-mice (all *P* < 0.05) (Figure 13A). The capability of cytokine secretion (IL-12 and TNF-α) was also inhibited at the case of XBPi-transfection (Figure 13B) (all *P* < 0.05).

**Figure 14:**
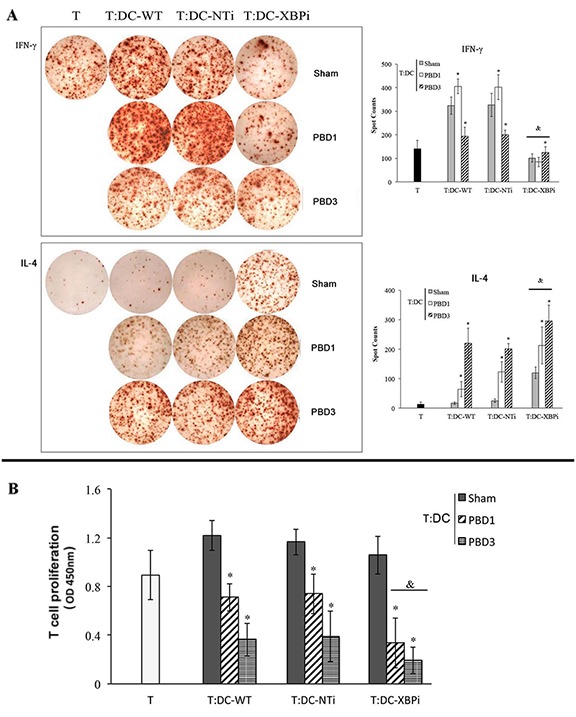
DCs from XBPi-burn mice could induce more notable hypo-responsiveness of CD4+ T Normal CD4 T (2*10^5^cell/well), after being stimulated with soluble CD3 (1 μg/ml) and soluble CD28 (5 μg/ml) for 24 hours, were co-cultured with splenic DCs isolated from XBPi-burn mice model for 3 days at a DC: T ratio of 1: 200 cultured. Production of IFN-γ and IL-4 were assessed with ELISpot Assay. T cell proliferation was detected with CCK-8 cell counting kit. Results were shown in mean ± SD (*n* = 6). * Statistically significant difference when compared with WT-sham group (*p* < 0.05). & Statistically significant difference when compared XBPi with WT or NTi group at the same time points (*p* < 0.05).

DCs from XBPi-burn mice could induce more notable hypo-responsiveness of CD4^+^ T cells and promote Th2 polarization than that from NTi-burn mice or WT-burn mice. As shown in Figure 14, co-culture with DCs from XBPi-transfected mice resulted in the inhibition of T cell proliferation, together with the marked inhibition of IFN-γ production (Th1-type cytokine), while with significant promotion of IL-4 secretion (Th2 type cytokine) (all *P* < 0.05).

## DISCUSSION

Severe suppression of the immune system is the major causative factor of infections following thermal injury. The mechanism responsible for initiating and controlling this immunosuppressive response after burns remains to be elucidated. DCs are known to play a key role in linking the innate and adaptive arms of the immune system. It was proved that there was a functional impairment of DCs following severe thermal injury, skewed toward an immunosuppressive phenotype with an impaired ability to activate T cell response [10–13, 25]. Consistent with these reports, the expression of maturation markers on the surface of DCs in the present study were shown to be down-regulated after burn injury, reflecting an impaired maturation of DC. Meanwhile, we noted a significant apoptosis in splenic DCs after burn injury, and it served as a hallmark of immune suppression.

To further investigate the mechanisms with regard to immunosuppression in major burns, the involvement of ERS response in dysfunction of DCs was focused in the current study. ERS is the endogenous self-protective mechanism, and it plays an important role in almost every process of living by regulating the balance between homeostasis and apoptosis. A series of reports have provided insights into the function of ERS response in the immune regulation. Many anti-stress pathways utilized by DCs are reported to be linked to the global regulation of protein synthesis and depend on ERS response [26, 27]. Results of our previous study have also proved that ERS is of great importance in the process of maturation and activation of DC *in vitro* [23]. In the present study, data revealed that there was notable ERS in splenic DCs following scald injury, showing an obvious up-regulation of GRP78 and sXBP-1, which are two important markers and key regulator of ERS response. ERS response in DC in our mice model of severe burn injury was shown to confirm our expectation. We further treated burn mice with Sal to counteract ERS-induced cell injury. Sal is reported to play a part in the enhancement or prolongation of eIF2α phosphorylation and reduces global protein synthesis as well as the overall amount of ERS, thus to be cytoprotective and beneficial to cell survival [24, 28, 29]. Data from this study showed that administration of Sal, especially at 6 h after burn injury, resulted in a significantly amelioration of inflammatory reaction and a markedly decreased mortality of burn mice. The down-regulation of GRP78 expression and XBP-1 activation after Sal-treatment revealed a correlation between the protective effect of Sal and the reduction of ERS response in DCs. Results of the present study demonstrated that treatment with Sal achieved better effects when it was administrated at 6 h than used at 1 h post-burn. However, the exact mechanisms of Sal protection on burns are not clear. Interestingly, our previous study had proved that the most remarkable suppression of T lymphocytes was at 8 hours after burn injury [11]. We hypothesized that Sal administration might modulate the ERS response at an appropriate juncture when it was administrated at 6 h post-burn. It remains to be further investigated how timing of Sal administration affects the overall control of immune dysfunction in severe burn injury.

The focus of our research is to assess the influence of inhibition of excessive ERS response on the host immune status. As apoptosis of DC is one of the main causes of immunosuppression in extensive burns, we investigated the effect of Sal-treatment on apoptosis of DCs. And because the functional properties of DC depend on its maturation status and phenotype [30], we also investigated the functional phenotype of DCs in burn mice treated with Sal. Treatment with Sal resulted in decreased apoptosis rate of DCs in burn mice, and up-regulation of the expressions of CD 80, CD86, and MHC-II on the surface of DCs. That is to say, Sal-treatment is able to alleviate the severe ERS response in burn mice, thus contributing to the regulation of DCs, thereby rendering phenotype maturation of DCs toward immunostimulatory response. Actually, splenic DCs from Sal-treated burn mice were demonstrated to have acquired a mature phenotype and became capable of antigen presentation and T cell stimulation. These were corroborated by the promoted T cell proliferation and polarization towards Th1 immunity. Taken together, the data support the notion that Sal might modulate ERS response and subsequent dysfunction of splenic DC induced by thermal injury, in turn protecting mice against major burns. Our findings demonstrated the important effect of Sal in abating severe injury-induced immune dysfunction of DCs.

Isolated two decades ago in a search for regulators of MHC-II gene expression, the XBP-1 is proved to be a key factor in the ERS response [31, 32]. Upon ERS, XBP-1 is spliced by IRE1, then functions as an important nuclear transcription factor by translocating into the nucleus to initiate transcriptional programs that regulate a subset of ERS-associated genes involved in the pathophysiological processes of various diseases. Transcription factor XBP-1 is reported to be essential for the development and survival of DCs [21], and it is also the key regulator in the process of activation and maturation of DCs [23]. To further investigate the significance of XBP-1 signaling pathway in the immunomodulation following acute insults, XBPi-transfected mice were subjected to burn injury in the present study. Our findings showed that XBPi-transfection had a negative impact on the capability of mice to withstand a severe thermal injury, resulting in higher mortality than normal or NTi-mice. XBPi-transfection led to a down-regulation of expressions of costimulatory molecules on surface of DC in normal mice. When XBPi mice were subjected to scald injury, these molecular markers on surface of splenic DC were slightly up-regulated but still lower than that in normal or NTi mice. Moreover, XBPi-transfection impaired the ability of splenic DCs to activate T cell effectively, resulting in the inhibition of T cell proliferation and polarization towards Th2 immunity. Further investigation revealed that XBPi transfection led to more significant apoptosis of splenic DC when the mice subjected to major burns. A notable up-regulation of CHOP expression and caspase-12 activation in DCs from XBPi-mice supported the notion that XBP-1-mediated adaptive ERS might be out of balance and bias toward apoptotic response. These results supported a pivotal role of XBP-1 in the maturation and immunomodulation of DCs. XBP-1 appears to be important in the balance between adaptive and apoptotic ERS response, however, the underling mechanism of ERS in DC maturation is unclear due to the complexity of the mechanisms of immune regulation *in vivo*. And the precise signaling pathways by which the ERS response play roles in DC maturation and activation deserve further investigation.

In summary, severe thermal injury trigs excessive ERS response in splenic DCs, which might initiate the apoptosis and functional dysfunction of DC. XBP-1 plays a pivotal role in the maturation and immunomodulation of DC. Interventions to modulate ERS signaling pathways towards adaptive responses may therefore be an important checkpoint for protection against cellular immune dysfunction and might be valuable in exploring novel therapies for major burns.

## MATERIALS AND METHODS

### Medium and reagents

Collagenase D from Clostridium histolyticum and triton X-100 were purchased from Sigma, St. Louis, MO. Salubrinal (Sal) was purchased from Tocris Bioscience, United Kingdom. NycoPrep 1.077A was purchased from Axis-shield Co., Norway. CD11c^+^ (N418) MicroBeads were purchased from Miltenyi Biotec GmbH, Bergisch Gladbach, Germany. RPMI 1640, fetal calf serum (FCS), glutamine, penicillin, streptomycin, and HEPES were purchased from TianRunShanda Biotech Co., Beijing, China. The medium used throughout the experiment was RPMI 1640, supplemented with 100 U/ml penicillin, 100 μg/ml streptomycin, 1.5 mM-glutamine and 10% heat-inactivated FCS. Nondenaturing lysis buffer and protease inhibitor cocktail were purchased from Applygen Technologies Inc., Beijing, China. Purified CD3e (clone 145-2C11), CD28 (37.51) were purchased from BD/PharMingen, San Diego, CA. Antibodies used for Western blotting analysis to determine glucose regulated protein (GRP) 78, C/EBP homologous protein (CHOP), and anti-X-box binding protein 1 (XBP-1) antibodies were from Abcam, Cambridge, UK. Anti-caspase (cas)-12, anti-PERK, and anti-p-PERK was purchased from Cell Signaling, Danvers, MA. Amersham ECL™ advance Western blotting detection kit was purchased from Amersham Pharmacia Biotech, Uppsala, Sweden. Antibodies used for flow cytometry analysis, including fluorescein isothioctante (FITC) anti-mouse I-A/I-E antibody, phycoerythrin (PE) anti-mouse CD80 antibody, FITC anti-mouse CD86 antibody, as well as anti-mouse CD16/32 antibody (Fc blocker) were purchased from Biolegend, San Diego CA. ELISpot assays of interleukin (IL)-12, tumor necrosis factor (TNF)-α, IL-10, IL-4 and interferon (IFN)-γ were purchased from R&D Systems, Minneapolis, MN. Cell counting kit (CCK-8/ WST-8) was purchased from Dojindo, Kumomoto, Japan. FITC-conjugated goat anti-mouse IgG2A was purchased from Biosynthesis Biotechnology Co., Beijing, China. ELISA kit of high mobility group box-1 protein (HMGB1) was purchased from SHINO-TEST Co., Kanagawa, Japan. ELISA kits of IL-12 and TNF-α were purchased from Dakewe Co., Shanghai, China.

### Animals and surgical procedures

All experimental procedures were undertaken in accordance with the National Institute of Health Guide for the Care and Use of Laboratory Animals, with the approval of the Scientific Investigation Board of the Chinese PLA General Hospital (No. SYXK2012-018), Beijing, China. Adult male BALB/c mice (6 to 8 weeks old and weighing 19-21 g) were used for the experiments. All animals were housed in a temperature-controlled room with 12 hours (h) light and 12 h darkness to acclimatize for at least 3 days before being used. All animals had free access to water, but were fasted overnight prior to the experiment. Procedures for full-thickness scald injury were performed as described previously [11]. Briefly, under anesthesia with pentobarbital sodium *via* intraperitoneal injection, the dorsal and lateral surfaces of the mice were shaved, a protective template with an opening corresponding to 15% of the total body surface area (TBSA) was securely placed on their back, and the exposed skin was immersed in 95°C water for 8 s. Ringer’s solution (1.0 ml) was administered hypodermically for resuscitation 1 h after thermal injury. Sal (a selective inhibitor of eIF2α dephosphorylation [25]) was dissolved in DMSO (Sigma Aldrich, St.Louis, MO) and further diluted with saline. Mice of Sal-treatment group were given injections of Sal (0.1 mg/ml, 0.2 ml per mouse) intraperitoneally (ip) 1 h or 6 h after the insult. Mice of control groups were given normal saline (NS). Mice of sham group were subjected to all of the same procedures except for the actual insult.

### XBP-1 RNAi lentivirus generation and transfection *in vivo*

RNAi lentiviral vector (LV) was synthesized and provided by GeneChem Company (Shanghai, China). A short hairpin RNA (shRNA) designed against XBP-1 (XBPi) was constructed by inserting two complementary oligonucleotides for 5′-GGGATTCATGAATGGCCCTTA-3′ into the pGCL-GFP and cloned into pFIV-H1/U6-copGFP vector as in previous report [24]. A nontargeting stem-loop oligonucleotide was used as a sham-silence control (NTi). Six-week-old male mice were individually injected with LV (3.5×10^7^ TU per mice) *via* the tail vein. After transfection for two weeks, transfection efficiency of LV in DCs *in vivo* was monitored by fluorescence microscopy after isolated with CD11c^+^ MicroBeads (Miltenyi Biotech, Bergisch Gladbach, Germany), roughly 60% of splenic DCs were transfected with LV.

### Purification and isolation of splenic DCs and T lymphocytes

Under aseptic technique, spleens of mice were harvested and washed with ice-cold PBS for two times. Mononuclear cells were isolated from collagenase D-treated spleen preparations with NycoPrep 1.077A density gradient medium as previously described. Splenic DCs (CD11c^+^) and T lymphocytes (CD4^+^) were isolated from mononuclear cells by MicroBeads and a MiniMACS™ Separator (Miltenyi Biotech, Bergisch Gladbach, Germany) with a positive selection LS or MS column following the manufacturer’s instructions. The isolated cells were pelletized by centrifugation (300 g, 10 minutes), the supernatant was discarded, and the cell pellet was resuspended in the desired volume of RPMI 1640 FCS (10%) medium, then cultured at 37°C in 5% CO_2_ in humidified air, or used for experiments.

### Western blot analysis

The sample of spleen (50 mg) or isolated DCs (10^6^) were collected and washed with cold PBS, and lysed with lysis buffer (150 mM NaCl, 1.0% NP-40 or 0.1% Triton X-100, 0.5% sodium deoxycholate, 0.1% SDS, 50 mM Tris-HCl pH 8.0, Protease Inhibitors). After incubation on ice for 20 minutes (min), the homogenate was centrifuged at 12000 rpm for 25 min at 4°C, and protein content in the supernatant was measured using a Bradford protein assay kit. Thirty micrograms of total protein of the supernatants was boiled at 96°C for 5 min after mixing with SDS-loading buffer, and was separated with 8-10% SDS-PAGE (Pulilai Co., Beijing, China) and transferred to nitrocellulose membrane. Specific antibodies were used to detect GRP78, XBP-1 [both full-length XBP-1 (unactivated, uXBP-1) and spliced XBP-1(activated, sXBP-1)], CHOP and cas-12 [both unactivated caspase-12 (Ucas-12) and activated cas-12 (Acas-12)]. A monoclonal anti-β-actin antibody (Sigma Aldrich, St. Louis, MO) was used as a control for protein loading. Immunoblots were visualized by enhanced chemiluminescence (Amersham Biosciences, Uppsala, Sweden). The blots were stripped in stripping buffer at 37°C for 45 min, washed twice with TBST buffer for 15 min each, and re-probed with another antibody. Representative images from the same membrane were selected and quantified for the abundance of interest proteins by densitometry (Bio-Rad Laboratories, Hercules, CA).

### Phenotypic analysis of DCs

DCs (5×10^5^) were reacted for 15 min at 4°C in 100 μl of PBS 5% FCS, 0.1% sodium azide (staining buffer) with PE-conjugated IgG specific for CD80 and CD86, and FITC-conjugated IgG specific for major histocompatibility complex (MHC)-II. In all experiments, isotype controls were included using an appropriate PE- or FITC-conjugated irrelevant monoclonal antibody of the same Ig class. Cells were then washed twice with PBS 5% FCS, 0.4% paraformaldehyde in PBS (pH 7.2-7.4), and examined by flow cytometry using a FACScan (BD Biosciences, Mountain View, CA).

### The apoptosis of DCs

The percentage of apoptotic DCs was determined with annexin-V-PE/7-ADD co-staining assay according to instructions provided by the manufacturer. In brief, isolated splenic DCs (5×10^5^) were resuspended in 100 μl binding buffer, to which 5 μl Annexin V-PE and 5 μl 7-AAD were added. After incubation for 15 min in darkness at room temperature, the cells were diluted with 400 μl binding buffer and analyzed by flow cytometry within 1 h using a FACScan (BD Biosciences, Mountain View, CA).

### T cell activation and proliferation assay

T cells isolated from normal mice were plated in 96-well plates at 2×10^5^ cells/well, cultured for 24 h in complete culture medium containing soluble CD3 (1 μg/ml) and soluble CD28 (5 μg/ml), then mixed with DCs from burn mice model at a DC: T ratio of 1: 200, or without DCs, and cultured for 3 days. The proliferation rate of T cells co-cultured with DCs was assessed with CCK-8 according to the manufacturer’s instructions. CCK-8 (10 μl/well) was then added and incubation was continued for 2 h. The optical density was measured by the use of a microplate reader (Spectra MR, Dynex, Richfield, MN) at the wavelength of 450 nm.

### Cytokine secretion measured by ELISpots

Secretion of cytokines including IL-12, TNF-α, IL-10, IL-4, and interferon (IFN)-γ was determined with ELISpots, strictly following the protocols provided by the manufacturer. In briefly, the isolated DCs from thermal injured mice were resuspended with RPMI 1640 FCS (10%) medium and plated to the microplate alone (2×10^5^/well) or co-cultured with T cell as described above, then cultured at 37°C in 5% CO_2_ in humidified air for a favorable period of time. All samples and controls were assayed in 4 wells according to the procedure and analyzed with ELISpot reader system (Spectra MR, Dynex, Richfield, MN).

### Statistical analysis

Data were represented as mean ± standard deviation (SD), and analyzed with a one-way ANOVA. Unpaired Student’s *t*-test was used to evaluate significant differences between two groups. The survival distributions of burn mice were compared with the log rank test. A *P*-value < 0.05 were considered statistically significant.
